# Remarks on Mr. Yeatman's Reply to an "Old Practitioner."

**Published:** 1822-05

**Authors:** Francis Bush

**Affiliations:** Member of the Royal College of Surgeons.


### MEDICAL ETHICS.

**Art. II.**—*Remarks on* Mr. Yeatman's *Reply to an* "Old Practitioner." By Francis Bush, Member of the Royal College of Surgeons.

"Qui odium in præcordis fovet, viperam amplexuro similis est."

In the Journal for July 1821, I submitted a paper, with the above signature, on the frauds practised to gain admission into the medical profession, which appears to have given some offence to Mr. Yeatman. I am not surprised at being a little bespattered on this occasion; for he who holds up an abuse to public notice, may reasonably expect that some delinquent will be hardy enough to step forward to defend it. The urchin who has played the truant, often betrays himself, by trembling at the sight of the rod; and I think Mr. Yeatman's morbid sensibility may induce some persons to suspect that his conduct has not been free from blame. In my former paper, it may be seen that I called the attention to what has been a frequent cause of complaint, and invited the co-operation of the members of the profession to guard our common interest and the public health. This I was induced to do, from having recently witnessed several persons gain admission to the profession by tortuous and irregular means: I will recite some of them.—A man, who, when I first knew him, was an illiterate servant to an attorney, and subsequently taken into his office to write: with this employment he soon joined that of druggist, having opened an obscure shop for that purpose. He continued this for a short time, and then disposed of the concern to a lad, who had been with him some two or three years; himself retiring into a village a few miles distant, and assuming the character and practice of surgeon-apothecary, where he continues to—shall I say—administer to the health of his neighbours, or increase their miseries by the unskilful use of medicines? *His pupil* soon gave up the druggist's shop, and engaged himself as assistant in some distant county, *(medicinam ignoto facere cæpisset loco;)* spent a winter in London; obtained a surgical diploma; and is now in full practice as surgeon-apothecary and man-midwife. I suppose these gentlemen were "favoured by the genius of medicine!" Your readers will form their own opinions of Professor Yeatman's anatomical lectures, and his system of education, as taught at Frome! and what will be the probable consequence of the introduction of *gentlemen* to the profession, armed with his certificates, and favoured by the genius of medicine! I think I might say,—

> "Scourges for sin," the spurious "tribe are sent;
> Like fire and storm, they call us to repent:
> But storms subside, and fires forget to rage;
> These are eternal scourges of the age."

The Apothecaries' late Act renders it illegal for any person to practise as an apothecary, or an assistant, who has not served an apprenticeship of five years, and submitted to a subsequent course of study. This Act having passed so lately, (an abstract of which may be found in the 34th volume of this Journal,) I cannot suppose Mr. Yeatman ignorant of it. The pupils he may have taken for a less term than five years, cannot become legally qualified for general practitioners, without some new arrangement. I would pardon delinquency arising from ignorance, but mark with severe censure any intentional evasion of the Act; and which, I think, Mr. Yeatman's reply shows he has been guilty of.

In the appendix to the reply, Mr. Yeatman has adverted to his case of amputation at the instep, in the 36th volume of the Journal; and, as his insinuation evidently refers to observations I made on it, soon after it was published, at a meeting of the whole of the medical practitioners of Frome, (he being present,) I beg to submit a few remarks on the case he has thus, after several years, dragged into notice; not as questioning the propriety of such an operation in some cases, but as entertaining doubts of its propriety in the case in question. There is an error in the first date, of some importance. The child was brought to Mr. Y. on the 28th of March, (and not on the 28th of September:) it had been submitted to the gentleman who had the care of the poor up to the 25th of that month, and he considered it a case of chilblains. From the 25th to the 28th, its history is unknown to me; but, on the latter day, Mr. Yeatman introduces it to public notice. His words are,—"Sarah Slade, a child four years old, was brought to me with gangrenous toes on the left foot; all the toes, and a small portion of the foot, of a jet-black colour, cold, and devoid of feeling; a slough, of the size of a shilling, under the heel; and, on moving it aside with the nail of my fore-finger, a point of the os calcis to be seen, denuded and black."

I would call the attention of my readers to the latter part of this sentence,—namely, that there was a slough on the heel, of the size of a shilling, which, on being moved aside with the nail of his fore-finger, (a clumsy substitute for a probe,) a point of the bone presented itself, denuded and black. I think a little attention to Mr. Yeatman's sequel will show that he was mistaken, and that it was not a "black and denuded point" of os calcis which he saw: every surgeon ought to know that a bone, when denuded and black, cannot furnish granulations till exfoliation has detached that portion; and, as the disease commenced only a few days before the 28th of March, exfoliation could not have been effected by the 8th of April, (in ten days,) at which time he states the wound to be nearly healed. Even on the 2d of April, (five days after she became Mr. Yeatman's patient,) "healthy granulations" were forming; and, on the 6th, "the appetite is tolerably good, and no symptom of irritation apparent." Mr. Yeatman's words are—"a small portion of the foot appeared to be affected," only; and yet he thought it necessary to dissect back the integuments, and remove all that portion of the foot anterior to the tarsal bones! From his own statement, it is clear he was mistaken in the state of the heel; and it is no very unfair inference, that he was mistaken as to the state of the toes also, and, as the local amendment was so rapid, the constitutional symptoms having disappeared, that the cure, if left to nature, would have been effected with *less deformity*, *less pain*, and *less danger*, than was incurred by the operation.

It is a commonly-received opinion, and I have acted on it through life, that, where an operation of any importance appears to be called for, unless the character of the disease be so urgent as to render delay dangerous, a second opinion should be taken. I would ask Mr. Yeatman whether or not he had the sanction of any other surgeon for the operation? If this question must be answered negatively, I will take the liberty to say that it was resorted to precipitately, if not unnecessarily.

About six years ago, John Cirtus, a nightly prowler, in his wanderings fell into a saw-pit near my house. He was brought into my surgery in the morning, at a moment when I was passing through it to attend some urgent call. I noticed the case superficially, but saw there was a wound of considerable extent on the thigh. I had not the care of the poor, but ordered him to be taken care of, from humanity only. Mr. Craddock, who was then my apprentice, was requested to attach the edges of the wound by the interrupted suture, support the parts by bandage, and send him to the workhouse; where he was under the care of Mr. Yeatman, who was parish-surgeon for that year. If inflammation came on, it was *his duty* to cut the sutures, and apply such topical and general remedies as the case required.

Your readers will perceive *quo animo* Mr. Yeatman's appendix is written! Who is there that, on reading his contemptible insinuation, would not suppose I had been botching a lacerated hand or foot, instead of having merely sanctioned the employment of sutures in a wound of the most fleshy part of the thigh?

The case of diseased stomach, to which Mr. Yeatman refers, is one of considerable interest: *he can tell how he obtained the patient; what he supposed the case to be,* and *the medical treatment.* I attended only the examination *post mortem.* However, I took minutes of the appearances, and may be able to correct any inaccuracies into which he might be led in that part of its history.

As Mr. Yeatman felt sore at my former communication, I should not be surprised to find him a little ticklish again: but I shall take my leave of him on this subject, assuring your readers that I lament the necessity of having trespassed on your valuable pages; and him, that I shall guard the profession against the introduction of improper persons, by every legal and honourable means: and I trust my example will be followed by the profession generally.

*Frome; March 15th,* 1822.

